# Resurgence from Crisis Through Awareness of Natural Inclusion

**DOI:** 10.1007/s42087-022-00293-8

**Published:** 2022-06-18

**Authors:** Eva Vass, Alan Rayner

**Affiliations:** 1 School of Education, Western Sydney University, Milperra, NSW 2214 Australia; 2 Bath, UK

**Keywords:** Natural Inclusionality, Resurgence, Second perspective, Dialogic education

## Abstract

For millennia, the Western mindset has been predisposed to the inherited custom to split mind and matter, emotion and cognition, art and science, the spiritual and the intellectual, the inner and outer, contemplation and objective inquiry. Our contemporary sense of dislocation from nature has arisen from this objectivistic perception. The current societal, economic and environmental crises urge us to break down the pervasive conceptual boundaries and binary distinctions and rethink what science is or what it can become. Covid 19 has exposed the iniquities and deep fault lines in the political and economic structure of globalized modern society founded on false division or unification of individual from or with group. We see Natural Inclusionality as the path to begin to resurrect and cultivate a *new normal* of co-creative community from the wreckage of the *old normal* of cultural tyranny (Rayner, 2020). The most ground-breaking dimension of the NI perception lies in its rejection of both extremes of dualism: the narrow objectivistic view of the world as dividable into discrete entities and the equally limiting monistic view of the world as some sort of uniform oneness. We propose to unpack these insights through dialogue: an ongoing professional dialogue between two authors, between two philosophical orientations, between natural and social sciences and between science and art. Our intentions are to show Natural Inclusional awareness as humanity’s ultimate resource for a resurgence from crisis—breaching the *Great Lie* that isolates individual from common good. In particular, we discuss the significance of these considerations in the context of educational science and practice.

## Introduction

This manuscript has evolved from a recorded conversation between the authors which was presented at the Psychology of Global Crises Virtual Conference (May 20–30, 2020) hosted by the American University, Paris. As the abstract outlines, the conversational presentation introduced the principle of Natural Inclusion and related this principle to the planetary and human crises we have been experiencing. In this edited manuscript, we expand on our Natural Inclusional understandings of the crises with a discussion of key implications for theorizing, research and practice in the field of education. In doing so, it captures our ongoing professional dialogue, bringing the Natural Inclusional approach into confluence with educational theorizing.

This dialogic paper is philosophically oriented and, as such, does not rely on the unpacking of empirical data, neither does the conversational narrative constitute new data. Our primary goal is to seek and articulate philosophical understandings through dialogue and work with these understandings in the context of contemporary societal tensions and dilemmas. The paper provides insight into the evolution of such understandings through our conversational, transdisciplinary, mutually receptive-responsive inquiry. It thus does not represent a definitive closure but marks a dialectic moment of convergence. When we bring in empirical work, we do so to illuminate particular points in our dialogue and to exemplify how our independent research trajectories (in natural and in social sciences) have led to complementary and confluent realisations.

Natural Inclusionality is a philosophy and fluid boundary logic of self-identity and ecological and evolutionary diversity and sustainability. It is based on awareness of the fundamental evolutionary principle of ‘natural inclusion’: the mutually inclusive, co-creative, receptive-responsive relationship between intangible spatial stillness and energetic motion in the being, becoming and evolutionary diversification of all material bodies, including our own. In essence, it arises from the simple move from regarding space and boundaries as sources of discontinuity and discrete definition to sources of continuity and dynamic distinction (Rayner, 2018; 2017; 2011a). It is intended to supersede the abstract rationality that has dominated human thought for millennia, based on definitive logic that can only apply to inert material systems that are unknown to exist anywhere in nature. The Natural Inclusional approach was first developed by Author 2 in correspondence with others during 2000, when it was termed ‘inclusionality’ (Rayner, 1997, 2003, 2004). Whilst the immediate scientific context for this philosophical approach was biology, inter-disciplinary dialogue has revealed rich congruences with approaches to scientific inquiry contemporaneously emerging in other fields: transfigural mathematics (e.g. Shakunle, 2010), quantum physics (e.g. Marman, 2016), cognitive sciences (Rayner & Jarvilehto, 2008), embodied cognition (Johnson, 2017) and phenomenological approaches to dialogism (e.g. Vass, 2019, 2018).

## The Principle of Natural Inclusion

Eva: First of all, I’d like to ask you to briefly introduce Natural Inclusionality to our audience.

**Fig. 1:**
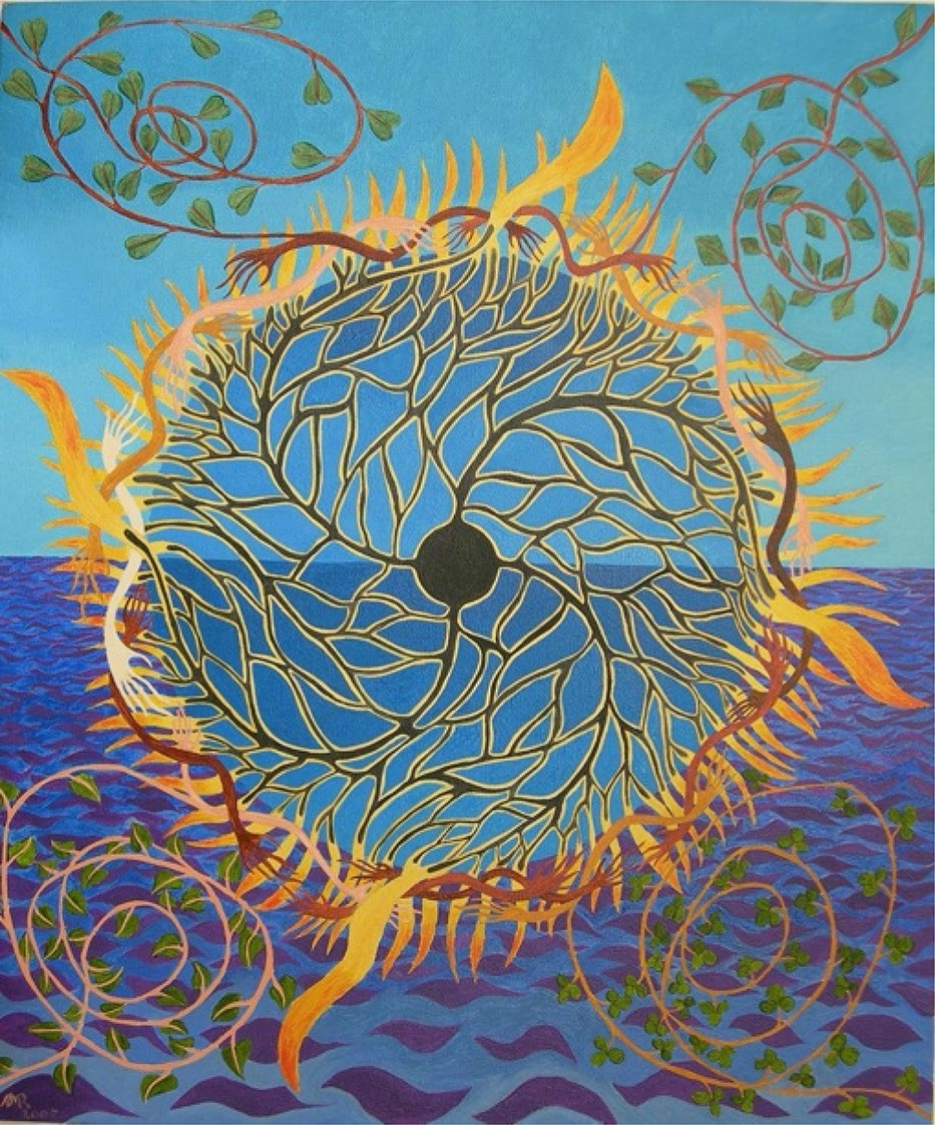
Holding Openness (Rayner, 2005)

Alan: Natural Inclusion to my mind is a fundamental evolutionary principle. It enables us to understand the true nature of reality as a varied expression of natural energy flow around and between local receptive centres of space. Now that’s a very short way of describing it which will take quite a long time to unpack and to understand. I could put it in another way; beneath the complex surface appearance of reality lies a simple truth… a dance between infinite receptive spatial void and local responsive energetic motion. Darkness and light co-creatively combined in myriad variations around a simple central theme. Perhaps you’d like to show at this stage an image of my painting *Holding openness*. That painting is celebrating this co-creative dance between darkness and light (Fig. 1).

This painting really sums up the basic understanding I have of Natural Inclusion in terms of this relationship… this receptive-responsive relationship between a centre of space, which calls energy into motion around itself. In this way we can understand how all material bodies – including our own human bodies – from subatomic scale outwards come into being and diversify as flow forms. We understand all material forms as flow forms – as mutual inclusions of void space and circulating energy in receptive responsive relationship^1^. And when we think about it, this is actually an expression of ancient spiritual wisdom in a modern scientific guise that appreciates the artfulness of all life on Earth. We understand ourselves and other beings as dynamic inhabitants of one another’s natural spatial and energetic neighbourhood… distinct identities together in receptive-responsive relationship, not independent entities set apart from one another and our surroundings. So that is essentially a very brief description of what Natural Inclusion is.

## Beyond Objectivistic Science Through Second Perspective

Eva: Alan, there is also an additional question here before we approach the current crises from a Natural Inclusional perspective. I would like you to characterize ways in which Natural Inclusionality either negates or goes beyond the mindset that we associate with objectivistic science. You are an evolutionary biologist, a natural scientist. But you are actually describing something bigger and more complex than what most of us would understand as the science of nature.

Alan: Yes, I’m going beyond the objectivistic view of nature that comes from a purely third-person approach to scientific inquiry^2^. Divorcing subject from object has become traditional in science: a way of viewing the world only from outside inwards, so our vision stops at the surface of what we observe and objectifies them. I’m also using an approach that speaks from within, so I’m essentially combining outside inward points of view and inside outwards points of view. If you like, I’m combining first-person subjective and third-person objective, viewing reality and putting those together in a creative way, which is like a second person point of view. This is where we actually have a more intimate understanding of what we’re observing in relation to one another. I’m using an empathic as well as an outside-in approach. And that’s very important, to bring empathy for whatever it is you’re observing into your understanding. This is not anthropomorphism, because anthropomorphism is just trying to put a human mind into the place of what’s being observed. This empathic approach is truly to imagine how it feels to be in the place of what you’re observing. And that’s an approach that I’ve actually always used throughout my biological research, in my studies of plant life, fungal life and so on. I’ve always used that approach and I’ve found that it leads to insights that are just not available when you adopt a purely objectivistic approach.

**Fig. 2:**
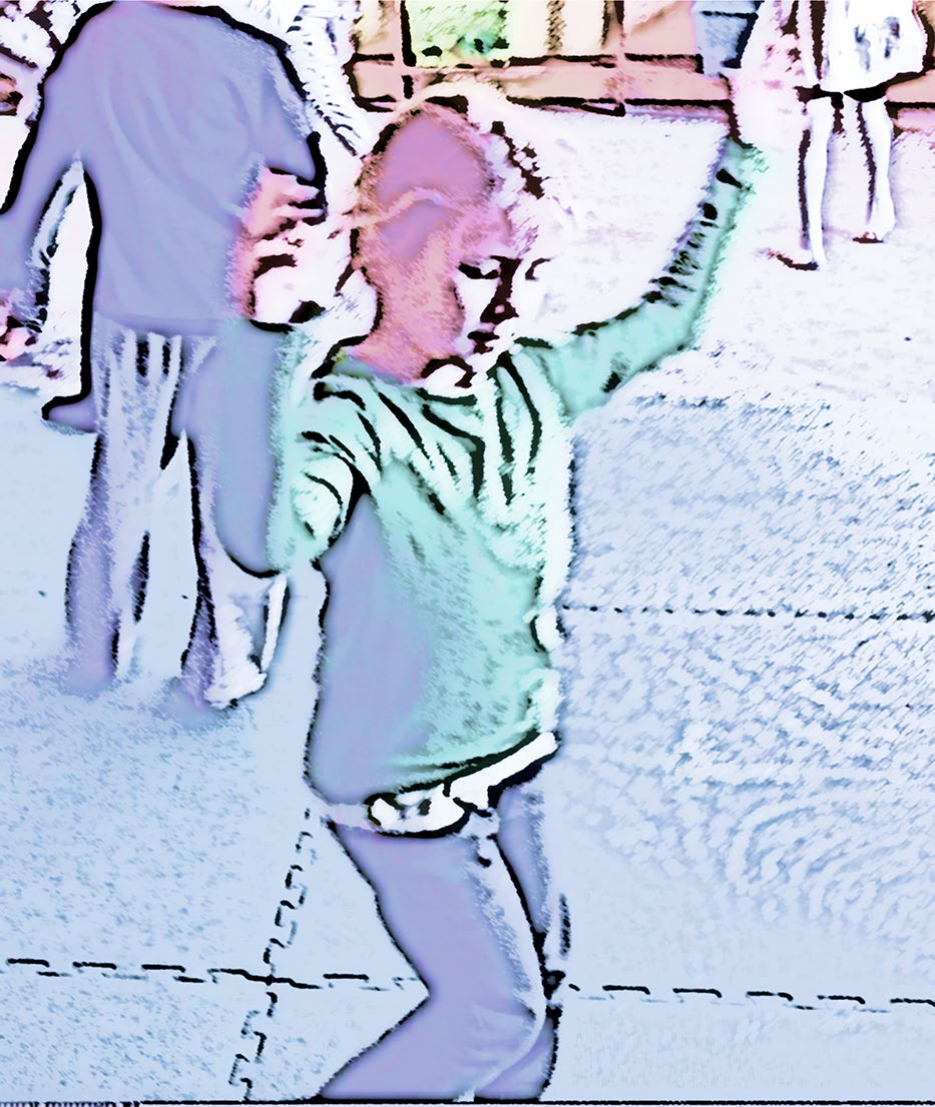
Musical encounters documented in movement (Vass, 2018)

Eva: This is a fundamental point, provoking deep ontological questions about the nature of reality and the way we experience ourselves as embedded in or extracted from our physical, natural, social context. This, in turn, impacts on epistemology and methodology: how we approach knowledge and how we understand scientific inquiry. I would like to illustrate these points with my reflections on my own research in music education. I frequently witnessed how young students develop deep musical affinity through free, active listening and movement improvisations (Vass, 2015, 2018). In these explorative music sessions somatic, lived insight was prioritised over structural analysis (Kokas, 1999). There is a clear distinction between such learning encounters and the factual, analytical knowledge building that dominates educational practice (Johnson, 2008, 2017). Whilst these two types of knowing are not distinguished in English, the German *kennen* (to know, to be familiar with) and *wissen* (to know a fact, know when/how) capture the distinction remarkably well. My research on experience-centred music education has provided powerful illustrations of musical learning as encounter. Expressions such as *hiding the music into one’s body* or *wrapping the body around the music* were used by child-participants to explain their intimate, transformative musical experiences (Vass, 2018; Kokas, 1999) (Fig. 2).

Whilst one may argue that musical knowledge is inherently embodied and lived (and so understanding music is like knowing a person), music education at large has its predominant focus on the science of music and on skills training (Kokas, 1999). There are a number of alternative approaches which counter this trend, with an emphasis on music appreciation and reception in early childhood (Huhtinen-Hilden & Pitt, 2018). These approaches share the fundamental principle regarding the inseparability of music and movement, with the consequent assumption that deep understanding of music inevitably goes beyond technical or theoretical knowledge and is enriched by musical experiences that are active and intersensory. The pedagogy I research was developed from the Kodaly philosophy of music education by Klara Kokas (1999), to guide children towards concentrated attention and an absolute perception of classical music through movement and creative work^3^.

Unsurprisingly, the adult musicians participating in my research often express resentment that their musical training may have severed their intimate relationship and natural affinity with music (Vass, 2016, 2019). I believe that these adult musicians are on a journey towards the same cathartic realisations as yourself, during your evolution as a natural scientist. Could it be that an empathic, phenomenological orientation is lacking in their own artistic and professional education? Could it also be that such phenomenological orientation towards learning and teaching empowers them to bring together the first-person perspective (introspection, focusing on inner sensations and feelings) and a third person perspective (looking outside, observing and interpreting the physical, musical and social space)? My research shows how collective creativity and imagination emerges from this dynamic interplay between the inner and the outer of the experience (Vass, 2019). But more importantly, it also reveals that we cannot move towards the wider acceptance of the second-person perspective as a legitimate approach to scholarly inquiry and intellectual discernment without an appreciation of embodied aesthetics of the mind (Johnson, 2008) that is grounded in a phenomenological orientation towards our lived personal experiences^4^.

I recall that you expressed these important continuities in your own teaching in higher education, bringing together students of biological sciences, natural sciences, management and psychology in collective, imaginative inquiry (see discussion in Rayner, In print).

## The Origins of Global Crises

 Eva: Let us now turn back to our concerns about global crises. How can we use a Natural Inclusional approach to understand the origins of the current global crises?

Alan: Routing that back to the origins of global crises my feeling is that all global crises, the ones that we are currently experiencing, arise from false or partially false culturally embedded perceptions of human nature and/or the nature of reality. Those false or partially false perceptions cause psychological, social and environmental harm. We’ve been teaching ourselves to think, to perceive the world, if you like, for thousands of years actually in a way that causes harm. And harm manifests in the crises that we’re observing, and it manifests all the more, the more that we have globalized particular ways of thinking, particular perceptions of reality which are actually false or partially false.

So just to give you an example of such a false perception, I could mention the perception that life is a competitive struggle for existence. That’s a widespread perception of reality encouraged by Darwinism and it actually arises from a purely objectivistic way of viewing the natural world from outside inwards without taking any account of the internal workings of what is being observed. And so you arrive at this false perception. Now if you then spread that perception far and wide through the culture, you teach it in schools, how is that going to make you behave?

If we are all going around with this perception that life is a competitive struggle for existence, how are we going to behave in relation to one another and in relation to our natural environment, our natural neighbourhood? I can tell you quite straightforwardly how it’s going to make us behave. It was epitomized by Richard Dawkins when he wrote the book *The Selfish Gene*. It will make us behave as selfish organisms purely putting our own self-interest first, without understanding that our self-interest necessarily embraces the community, the neighbourhood that we inhabit. So that’s an example. And we see, once those sorts of perceptions are deeply embedded, that we’re going to come across situations again and again and again that we can’t think our way out of, because we’re stuck with an attitude of mind that says, ‘this is the reality’. And we literally cannot begin to imagine a different reality.

Eva: We can see the harmful impact of this mindset in Western forms of institutional education, where competition and individual achievement are prioritised over collaboration and mutual enrichment. We pit students and schools against each other, inflating the value of individual performance and achievement, whereas our pedagogic focus should be on building mutually receptive-responsive relationships, encouraging other-orientation and nurturing co-creativity. What seems evident to me is that there is yet another key reflection point central to our discussion here. Your critique of objectivistic science resonates with what I understand as the foundations of dialogic theory (e.g. Bakhtin, 1981). A Bakhtinian theoretical reframing can help us better understand the adverse effect of authoritative (monologic) teaching approaches which insist on the wholesale acceptance of a given way of thinking. It urges us to challenge the narrow understandings of substantive dialogue as the means to unify and homogenize our thinking, aligning it with, for instance, curricular objectives or expectations.

**Fig. 3:**
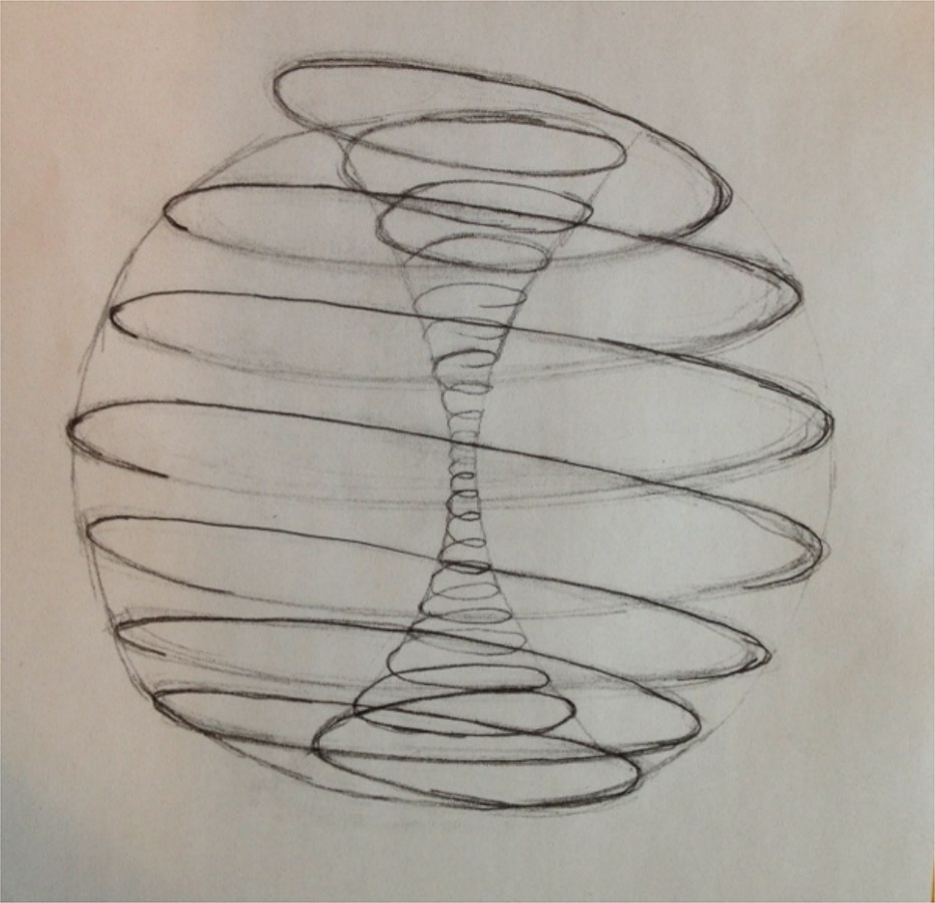
The vortex of perpetual dialogic flow (not the correct title) (drawing by Roy Reynolds, permission for reproduction granted)

Both NI and Bakhtinian dialogism show commitment to the being and becoming of life as essentially co-creative. From a Bakhtinian dialogic perspective inside and outside are inseparable, and a boundary is a place of encounter not separation. So, what I see especially important in both NI and dialogism is the understanding of boundaries as fluid and dynamic: a source of mutual enrichment and not separation. What you describe as mutually receptive-responsive relationships in nature, Bakthin sees as the self’s perpetual, dialogue with its particular physical, social or cultural environment. Our encounters with alterity generate dialogic tension and involve perpetual creative struggle as new ideas and potential understandings are formed (Wegerif, 2018). Whilst this flow grants us moments of resolve, it also anticipates the infinite, perpetual expansion of understanding without a predetermined point of ultimate closure. There is no endpoint to this process of dialogic expansion. I find the following image (Fig. 3) particularly powerful in illustrating this perpetual dialogic flow.

The congruence that I see between NI and Bakhtinian dialogism is important for me, both in terms of my research on music pedagogies and for education in general. It prompts both ontological and epistemological questions, re-imagining what knowledge is. I would argue that NI can indeed inspire further, transdisciplinary dialogic theorisations.

## Crisis as War

Eva: Let’s return to our overarching theme: the current crises. You talked about the harmfulness of a narrow objectivistic position and I think it would be really useful to apply it to the current situation. As we work our way through this crisis (the COVID-19 pandemic in particular) we describe it as a fight, as a competition, as a war. You are an expert of fungi you understand organisms that are often seen as harmful but not necessarily understood well. Is there a lack of understanding of what we are dealing with? Is this a false perception, seeing the virus as the enemy to fight against as opposed to seeking to better understand and manage it?

Alan: You know that’s exactly right. This is an attitude of the human mind that makes an enemy of the other and that leads us to go to war with what we perceive as an enemy… instead of deeply understanding the fundamental nature of what we’re dealing with or working with. So, one of the ways that I find that we can get out of that habit is to understand that all organisms, all living creatures are needful… That is how we get hungry, that can bring us into rivalry with others. It can be the case that our needs don’t coincide with others. But that doesn’t make us selfish, that doesn’t necessarily put us at odds with others. But we can understand one another’s needs, so you know a virus needs human cells to reproduce and that’s how it is. It is not at war with us, it is looking for a home in us. That is a very different way of thinking about what the virus is actually doing: it has found a home and what we actually have to do as human beings is say, terribly sorry, there’s no room here. I’m not letting you in, you need to go somewhere else. So, it’s very different.

There are many Natural History programs these days that are totally imbued with the idea of competition and warfare and that sort of imagery. But if we replace that with an understanding of need, we get a different feeling. Natural territoriality, parasitism, they are not the same as ideological conflict^5^. That is a purely human exercise, to actually make an enemy of the other. Does a gazelle regard a lion as its enemy? Does a lion eat a gazelle because it thinks the gazelle is inferior to itself? No, it doesn’t. So, there’s a very different way of thinking here. All organisms need to live. In order to live we have to take in energy from our neighbourhood, we can’t be otherwise. And the very fact that we need to take in energy that enables us to live means that we are not isolated from our neighbourhood. We cannot be isolated. It means that we are all living in one another’s mutual influence. One of the things about Natural Inclusion is that it takes us right deep down and extends down to the subatomic level, so that we can understand this relationship between the receptive need and the responsive flow of energy. And that’s chemistry.

## Alienation, Dislocation and the Great Lie

Eva: Linked to this, an idea that you often use is the notion of alienation. Alienation comes from the mindset of competition, and an understanding of life as survival of the fittest. Contrasting with that you often talk about the sustainability of the fitting. So, the loss of sustainability arises from the sense of alienation and the sense of dislocation of the self. Could you unpack this, and how it relates to what we are experiencing at the moment.

Alan: I put this in terms of what I relatively recently called the great lie. A number of people talk about the great lie: how we lie to ourselves about the reality of our own nature and about the nature of reality. And this great lie has the effect of severing or subsuming the uniqueness of individual self-identity from or within group identity and nature, that results in profound human conflict, oppression, psychological, social and environmental harm but it continues to be perceived and promulgated as literal truth by those holding, seeking or subservient to hierarchical power. So, we’re looking at power relationships here, and we’re looking at why we would be tempted, almost in a biblical sense, why we would be tempted to deny our human reality and the reality of the world that we inhabit. And ultimately, we may do that as a powerful combination of fear of the other, fear of death of course in human beings is very strong, fear of uncertainty is very strong and associated with that fear a kind of tunnel vision or partial view of reality. So that we deny an aspect of reality that we don’t want to admit. To put it in really fundamental terms, this is to my mind the most fundamental form of expression of the great lie. It is just this: tangible matter is either entirely separate or indistinguishable from intangible space. These two extreme perspectives are both an expression of the great lie. We think of matter as the material aspect of reality, space as the immaterial aspect. And we can either regard those two as being mutually exclusive, excluding one another or in opposition. We can speak of a battle between light and darkness, because that’s what we’re talking about here… and we often do. Thus dualism (or reductionism) isolates the material from the immaterial, treats those as never able to meet. Or [at the other extreme] we can say we’re all one, and we essentially try to remove any notion that natural forms can have boundary limits at all… can have constraining boundaries. There are fundamental issues with this extreme position of non-dualism (or holism): it does not make sense to conflate all reality as one whole reality in itself, removing all capacity for variation.

So, we get into this battle between philosophical dualism and philosophical non-dualism. And both of them are paradoxical, both are based on a partial way of viewing reality. Dualism comes from that objectivistic way of viewing reality, where you’re only looking from outside in. Non-dualism comes from looking only from inside out. But if you put the two together you see how they marry one another and give us the third way of Natural Inclusion, where we understand the mutual inclusion of energetic motion and receptive space within each other in the origin of all material form. Thus, space is recognised as a source of natural continuity, not an intervening distance^6^.

So that’s the great lie. And the effect that it has is if we just imagine all is one then we’ve essentially eliminated the idea that we have unique individual identities, we’ve killed our self-identity. If on the other hand we regard matter and space, the material and the immaterial to be mutually exclusive, then we’ve set up a battleground. We have dislocated our sense of self-identity and made it kind of an encapsulated, objective form against its surrounding. So that’s the origin of the idea of the struggle for life. We see the individual as a point mass, a unit of material which is at odds with the world that it inhabits. A friend of mine once described it as we make ourselves orphans from our natural source… and that’s exactly what happens. We then behave in a disoriented way, at odds with where we’ve come from and at odds with one another.

## Orphaned from its Source

Eva: This idea of ‘orphaned from its source’ powerfully captures the helplessness of objectivistic science… the narrow scientific mindset that is responsible for the problems that we are facing at the moment. How could objectivistic science possibly find the solutions to the problems that it generated? Now is there an image that we could use to illustrate your response to this question?

**Fig. 4:**
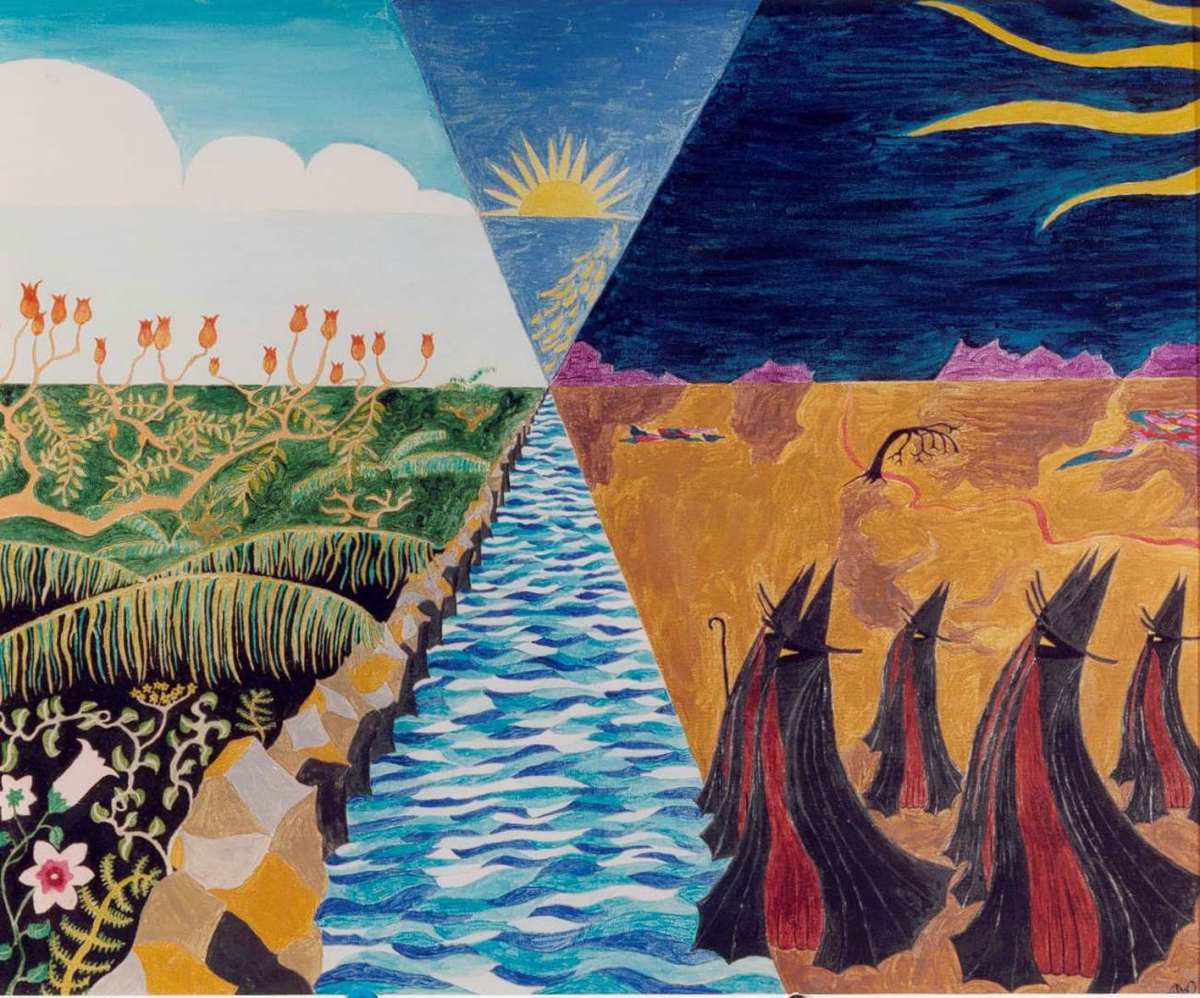
Arid confrontation (Rayner, 1973)

Alan: Yes, I think there is a series of images that are paintings that I made long ago when I was in my 20s and it was during my PhD research. The images illustrated my feelings quite strongly. If you can go to the image called Arid confrontation. That’s a painting that I made after a year of doctoral research (Fig. 4).

You know I was a naturalist, I was in love with the natural world, and I wanted to understand the world that I was observing, but I had been taught to practice science in this objectivistic way which had the effect of cutting me off as the cloaked observer from what I was trying to understand as the observed. And there was a barrier, a massive barrier of construction in the way of actually immersing my understanding in the world that I wanted to understand. And the feeling of desolation – the loss of soul is what *desolation* means, de-sol-ation – that was associated with the objectivistic way of viewing the world and not having any feeling for the interior life of what you’re observing. So those alienated creatures on the right-hand side of this screen, they’ve cut themselves off and they’ve made a Euclidean slash between themselves. And the Sun is represented as Euclidian circles and triangles, and so on^7^. But essentially that is a painting of alienation, literal alienation resulting from third person only perception.

**Fig. 5:**
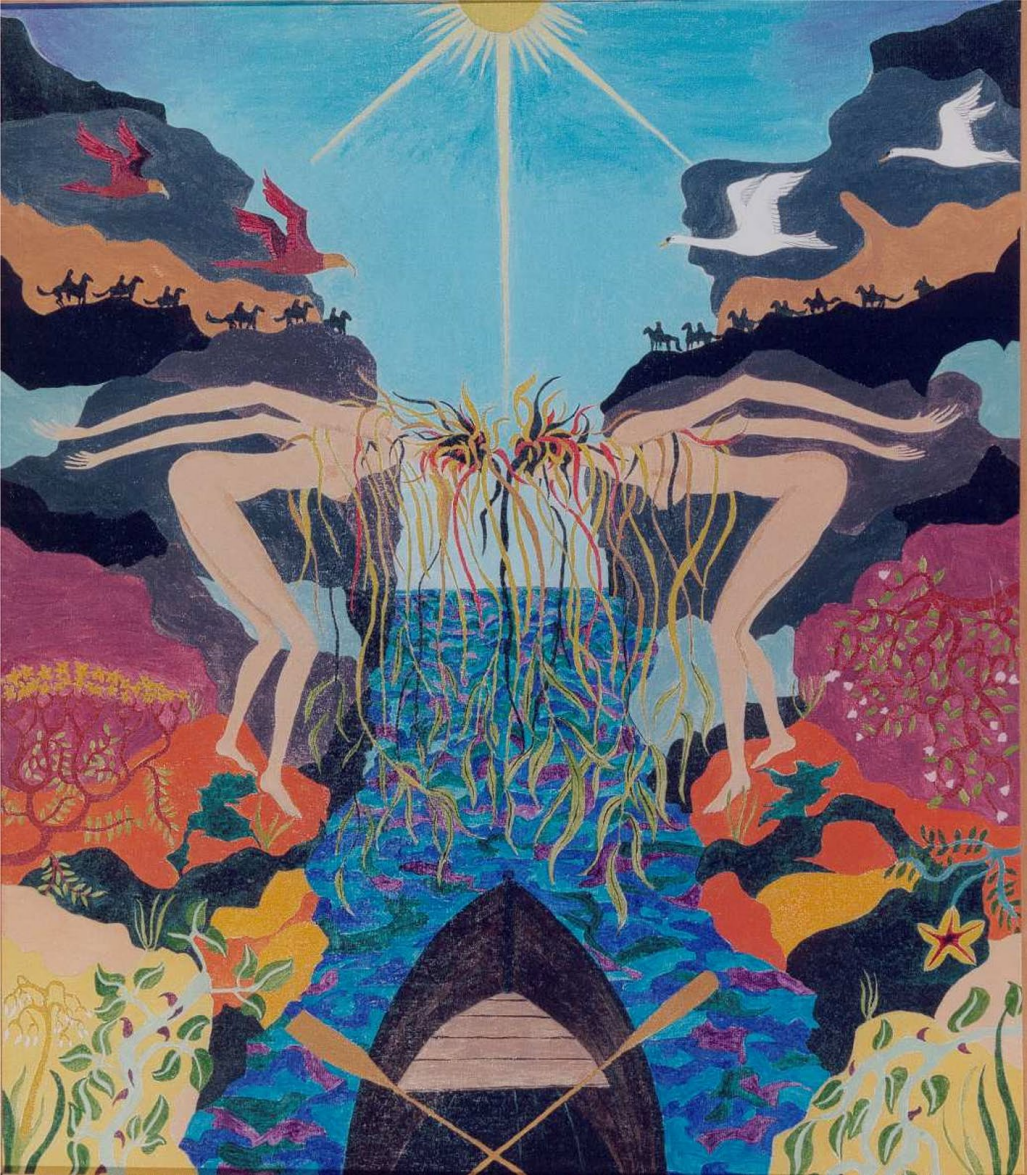
Willowy Bridge (Rayner, 1974)

Eva: I’d like to comment on this from my perspective. In the past we discussed this perception of alienation as an illusion and not necessarily reality. Such sense of dislocation or alienation is simply an illusion arising from the perspective itself. The reality is that you are deeply, inherently embedded and you cannot remove yourself even if you wish. But the removal of self through the third-person perspective creates that sense of alienation and creates that pain, struggle and incoherence. This is the image that you chose to represent these ideas (Fig. 5).

Alan: Yes, *Willowy bridge* is a painting I made a year later, when I was in my second year of research. It’s really about the relationships between the two different kinds of perception and the chasm that can open up between them but also how we can find the middle way between, that brings both into relationship with one another. So we see an empty boat, with crossed oars, and we see that the boat is making its passage through a veil out into the wide wide blue yonder, the open, through a willowy bridge of two female figures who are bridging two worldviews together. A worldview on the left which happens – and I had no idea that I was doing this at the time, it’s purely unconscious – happens to coincide with the left hemisphere of the brain and is associated with a strongly analytical view of the world, represented by hawks as predators with binary vision. That biological vision which gives a very strong focus on what is being observed and essentially objectified by the powerful outside-inwards view. And that is contrasted on the on the right with swans which have eyes on the sides of their heads and have a panoramic vision all around.

So, if you like, on the left we have reductionism and on the right so we have holism. Or on the left we have dualism and on the right, we have non-dualism. The empty shell of the boat, representing the soul, it’s making its way through. It’s negotiating its way between these seemingly opposed points of view and bringing them into a mutual partnership, instead of setting them at odds. We see that the sun here has a ray coming down, which is liable to cut through the bridge and divide those worlds again. It’s an extraordinary painting, I never understood it at the time. But as I looked at it more and more over the years since, I see it as a symbol of the journey that I was actually making towards Natural Inclusion. Which brings those two seemingly opposed world views into (a) dynamic, co-creative relationship with one another. So it’s quite a deep painting. It came as an answer to the alienation a year later.

## Methodology and Natural Inclusionality

Eva: Let us now turn to the significance of this Natural Inclusional approach next. You talked about the idea of resurgence and at this time of quite strong and deep sense of hopelessness. I am wondering how Natural Inclusionality can actually bring that hope, bring those new understandings/vistas that are needed in order to rise above and resurface. And I’d like you to address this question.

Alan: You know just recently I wrote this down. And this is why I do see hope and I do see the possibility of resurgence. I see that when we have recognized the deep falsehoods and divisions that reside behind our global crises, of which the current virus is merely a symptom, the spread of the virus is associated with over-networking essentially literally as a product of globalization in many ways, and the economic systems that we have concocted… and all those sorts of things which actually are going over to that ‘we’ve got to all be connected’ kind of attitude, in one in one mass… rather than actually recognize that there are good reasons for not over-connecting. You know real biological and ecological and evolutionary reasons for not doing it. So that is just one example. So, when we can actually begin to see what has been lying behind so many of our difficulties and we also begin to experience – as many of us have experienced in this period of astonishing calm within the storm – where we suddenly hear the bird song, we suddenly have unpolluted skies and we think what on earth have we been doing. Isn’t this now a time to reflect, reconsider and understand how we got into this difficulty.

That’s where my hope resides, because I’ve always felt that human nature is fundamentally loving, truthful and extraordinarily imaginative. And it is those qualities that reside at the heart of our individual and collective creativity and ability to learn. Those are the qualities that are our greatest human asset. That we can be misled to believe and to behave otherwise is due to that powerful combination of fear and partial perception which manifests in what I call those great lies that has become so deeply culturally embedded. The only way to escape the influence of that falsehood is literally to educate ourselves out of it. And that’s really what that last painting of mine was doing. And it’s a demonstration of leading yourself out of the conflict that arises from a false a false dichotomy between worldviews which are based on partial perceptions but are actually complementary.

I think it also shows, for me as a scientist… I’ve always practised art and it shows me that you know you cannot argue your way out of it. You cannot rationalize your way out of these crises. But art, in all its forms, offers us an approach, an educational approach especially, that can enable us to lead our way out of the ‘whole’ (with the W) that we’ve entrapped ourselves within. We can lead our way out of this entrapment and that is where the resurgence comes. That is where the hope, in my mind, lies. The recognition of the need both for individual nonconformity and for collective coherence. Not one or other in opposition but both in co-creative relationship.

So yeah, there we are. And that I think relates so very strongly to your own work. Your work in primary schools and in higher education. That is where you have been coming from in your pedagogy. I think you knew it all along. But it’s also right at the heart of the discomfort that you have felt in an educational system which is teaching us the great lie.

Eva: Indeed, an educational system that is impositional as opposed to catalytic and explorative^8^. It is evident to me that Natural Inclusionality has deep implications for the study of the mind as inherently embodied and dialogic. The natural mind has an inherent disposition to engage in a receptive-responsive dialogue with its natural and human environment. However, a major concern arises from the NI positioning. Does education – with its reliance on objectivistic perception – actually work against the natural orientation of the mind towards thinking from presence? Does the narrow focus on objectivistic perception dislocate us, severing the self from the world? If so, the ramifications are significant, and they go far beyond the walls of our classrooms. These questions have permeated my research throughout my academic journey.

In recent years I have been working with the notion of *receptive-responsive relationships* at different levels. I believe that this helps us to grasp the co-creative potentials of natural and social sciences (or sciences and arts) as dialogic partners, negating the either/or perception of objective science and subjective art. A Natural Inclusional approach encourages us to engage disciplinary fields in the same kind of receptive-responsive dialogue that we hope to promote in classrooms. What we see instead is fragmentation, a sense of dislocation which limits dialogue. Yet, objective and subjective perceptions can form a partnership towards comprehensive perception and insight. So why should our mode of inquiry focus on one or the other? From an observational point of view, you can observe and say what things ‘look like’ or appear to be but you can also examine your own lived experience of those things. Research thus becomes a ‘dance in between’ the insideness and outsideness of experience: a process which brings these into confluence.

**Fig. 6:**
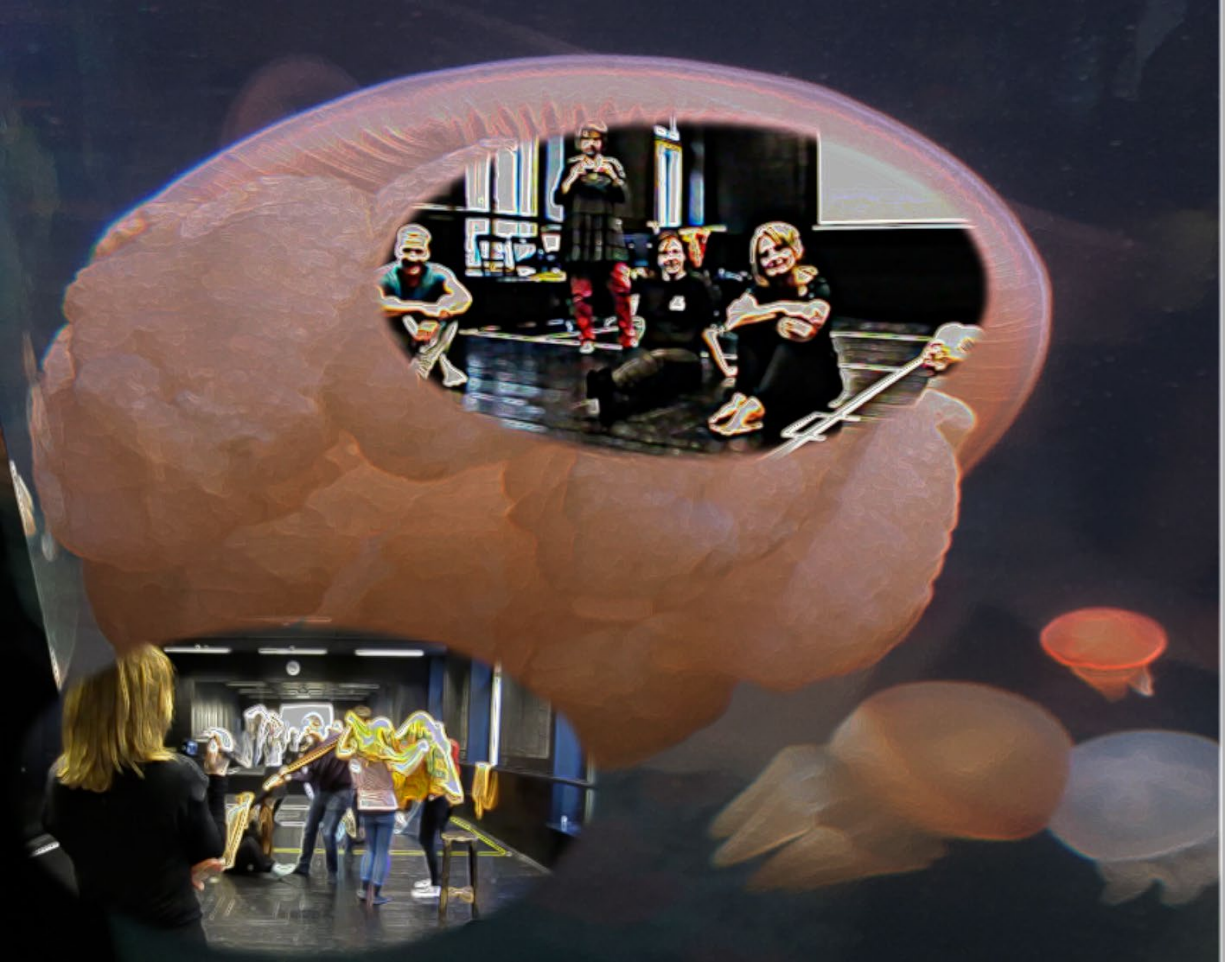
Researcher and researched

The following image illustrates my experimentation with these ideas. Whilst I am looking at my research participants (higher education students) they are also looking at me, the researcher. I am reflecting on my own felt experiences and I am also asking participants to share their introspective accounts, in talk or in writing (e.g. Vass, 2019). This way I can combine inward-looking inquiry with the outward looking, detached exploration of actions and insights. So my methodology works with the continuity between researcher and the researched (Fig. 6).

I think the second perspective is fundamental as a methodological framing, to grasp the true essence of the study of reality. This, to me, is a very important message for educational sciences.

Alan: Yes. And it’s also about being led from the heart. So you feel that receptive place in your heart. And when you make receptivity primary and allow that receptivity to bring all sorts of ideas of apparent conflict into confluence.

Author 1: And I guess that works at different levels in different contexts of inquiry. Returning to education, I like the idea of receptivity as a process of learning, a process of teaching or as the framing of other forms of inquiry at different levels. The idea of hope through a change of mindset but not necessarily simply shifting from one to the other but maybe bringing different perspectives together into confluence. So this is the significance of Natural Inclusionality in the midst of the crisis. This is how we can understand its value in resurfacing from the current sense of hopelessness or sense of loss. We explored and envisaged the necessary mindset change in this conversation and reflected on the consequent need to broaden the legitimised modes of scientific inquiry.

In closing, we present a poem by Alan Rayner:

**A Simple Message**

A simple message breathes into Mind.

Immerse your Self in the Receptive Stillness of Space,

Within Life,

Not aloof from IT.

Your innate creativity blooms,

Inspired and soothed

By Love

In the dark,

Soulful depth

Of your open heart.

## Footnotes

1 Natural Inclusionality builds on fluid geometry, understanding that nature is fundamentally fluid, and not digital. This entails the appreciation of the underlying spherical/circular geometry of natural forms and the recognition that curvature comes before linearity in the formation of natural material bodies. NI challenges conventional abstract geometry, which works with discrete boundaries demarcated by straight lines and which perceives the natural world in an overly definitive way. In mathematical and scientific terms, NI transforms the abstract Euclidean, non-Euclidean and fractal geometry into natural flow-geometry. It incorporates receptive space and intrinsic flux into mathematical figures as flow-forms. For further details on fluid or flow geometry, see Rayner (2018, In Print).

2 The issue of perspectives has been an enduring focus of philosophy. It is also an important epistemological issue, with different (first, second, or third person) perspectives regarded as different ways of epistemic access (see Pauen, 2012, for a valuable overview). The choice between – or the representation of – the first-person (subjective) and third-person (objective) perspectives has been debated in various approaches or paradigms of scientific inquiry. Considerations of the second-person perspective are, on the other hand, less widespread and often aligned with phenomenological orientations to science. See, for instance, Gallagher (2012) for comprehensive discussion of the ‘philosophical and interdisciplinary practice’ of phenomenology, where the participatory sense-making of the second (or ‘intersubjective’) perspective takes central stage.

3 The Kokas method is very similar to other creative, active music pedagogies in its core concepts. The most important distinction is that the Kokas sessions are not prescriptive or structured in any way, giving children almost infinite imaginative or creative freedom. Thus, the pedagogy breaks away from formulaic use of movement (e.g. instructing students to walk to the beat of the music), and it also goes beyond the structural analysis of music that is prevalent in traditional Western music education. Developed in the 1960s, it therefore foreshadows twenty-first century concerns and practices in Kodaly-inspired music education (Nemes, 2019).

4 Research in improvisative art contexts is rich in explorations of co-creativity. Concepts such as creative attunement (Seddon, 2004), group flow (Sawyer, 2015) or collective entrainment (Clayton, 2007) have been introduced in this research field to capture the emotional, intuitive, embodied essence of co-creation amongst adult artists. Author 1’s research moves from creative ‘production’ to creative perception of music. It explores a creative music pedagogy which openly invites and builds on such embodied co-creative experiences amongst young learners.

5 Rayner expands on this issue in their book (Rayner, 2017), detailing how the Darwinian notion of the ‘survival of the fittest’ – the idea that life is a win-lose competition between opposing entities – is not more than an invention of abstract perception. It has very little to do with the rivalry (natural territoriality) evident in nature. Contrary to the sense of ruthlessness in the abstract notion of competition, rivalry in nature shows receptivity and responsivity to the self and the other/neighbourhood. Thus, agility to shift from rivalry to alliance (a symbiotic relationship) or to yield to the other is just as vital as the capacity to gain from others.

6 Author 2 illuminates the path from both dualism and non-dualism as follows: ‘Instead of thinking about ourselves and others as either absolutely separate or inextricably connected, we recognise all forms as variably open to one another’s energetic influence. Our boundaries are energetic interfacings that make us distinct, as natural flow-forms, but not discrete, as independent singletons. There is no need to claim paradoxically that on the one hand there are “bounded wholes” whilst on the other “there are no boundaries between them”’ (Rayner, 2011b, p. 21). Heraclitus, the pre-Socratic philosopher, appears to be more relevant and valid than Democrates and Aristotle when we approach organic evolution this way: the dualism of the dual and the non-dual.

7 Please see the earlier commentary on abstract geometry versus NI’s flow geometry.

8 Dialogic approaches challenge such traditional teacher-directed, monologic, transmission-oriented educational practices. Resonating with Freire’s notion of the ‘pedagogy of the questions’, dialogic models facilitate collective meaning-making and open-ended educational inquiry through carefully managed, creative classroom dialogue. From this angle, the conversational sharing of rich and diverse perspectives (including marginalized voices and opinions) is the catalyst of deep learning. This underlines the importance of authentic dialogue amongst students and teacher, be it face-to-face or virtual, spoken or written. The conversation goes beyond didactic transmission of information or knowledge. It also goes beyond the competition around winning or losing an argument. Instead, it is active, inquisitive, empathetic and relational – serving mutual enrichment (Vass, 2017; Wegerif, 2018).
